# Effect of focused ultrasound cavitation augmented with aerobic exercise on abdominal and intrahepatic fat in patients with non-alcoholic fatty liver disease: A randomized controlled trial

**DOI:** 10.1371/journal.pone.0250337

**Published:** 2021-04-28

**Authors:** Mona Mohamed Taha, Yasser M. Aneis, Heba Mohamed Mohamady, Alrasheedy S. A., Shereen Hamed Elsayed

**Affiliations:** 1 Department of Rehabilitation, College Of Health and Rehabilitation Sciences, Princess Nourah Bint Abdulrahman University, Riyadh, Kingdom of Saudi Arabia; 2 Department of Cardiovascular/Respiratory Disorder and Geriatrics, Faculty of Physical Therapy, Cairo University, Giza, Egypt; 3 Department of Basic Science, Faculty of Physical Therapy, Cairo University, Giza, Egypt; 4 Department of Surgery, Faculty of Physical Therapy, Cairo University, Giza, Egypt; 5 Department of Internal Medicine, EL Sahel Teaching Hospital, Giza, Egypt; Kaohsiung Medical University, TAIWAN

## Abstract

**Objectives:**

The study aimed to examine the effect of focused ultrasound cavitation augmented with aerobic exercise on localized abdominal and intrahepatic fat in fatty liver patients.

**Methods:**

34 fatty liver patients aged 30–45 with a body mass index (BMI) of 30–40 kg/m^2^ were randomly assigned into two equally numbered groups. Group A received focused ultrasound cavitation and moderate aerobic exercise for three months, while Group B (control group) received moderate aerobic exercise only. Abdominal subcutaneous fat volume, visceral fat volume, liver-to-spleen ratio (L/S ratio), body weight, BMI, and waist circumference were measured both before and after the study period.

**Results:**

Both groups showed significant improvements in subcutaneous fat volume, visceral fat volume, body weight, BMI, and waist circumference relative to baseline where *(P < 0*.*001)*, with a higher percentage in group A. L/S ratio only showed a significant improvement in group A. Between-group differences were noteworthy regarding L/S ratio and waist circumference where *(P < 0*.*0001)*.

**Conclusion:**

While substantial risky measures in non-alcoholic fatty liver disease have been modified by aerobic exercise, its combination with focused ultrasound cavitation causes more notable effects on the reduction of abdominal and intrahepatic fat, making it a superior option.

**Trial registration:**

ClinicalTrials.gov: NCT04161703

## Introduction

Non-alcoholic fatty liver disease (NAFLD) is characterized by an increased and abnormal lipid accumulation intracellularly in the liver, mainly in the form of triglycerides [1]. In developed societies, the prevalence of NAFLD is nearly 20–30%, and 70–90% among individuals with diabetes or obesity [2].

According to the accumulated evidence, obesity is closely linked to an elevated risk of metabolic diseases, such as NAFLD [3]. Several studies demonstrated that visceral adiposity is a clinical predictor of hepatic steatosis [4, 5]. Hepatic steatosis is more predicted by **v**isceral fat than subcutaneous fat accumulation or body mass index (BMI) [4]. Therefore, assessment of visceral fat is important in clinical practices [6].

For assessment of NAFLD, lower liver to spleen ratio (L/S ratio) is considered to be a reliable index [7]. A recent study demonstrated that liver attenuation measurement on unenhanced computed tomography (CT) scans is the simplest and best method for predicting pathological liver fat content. Moreover, computed tomography is validated as a reliable, accurate, and quantitative measure of visceral and subcutaneous fat size [4].

Many modalities have emerged as methods of non-invasive reduction of adipose tissue, including high-intensity focused ultrasound (HIFU), low-level laser, and radiofrequency. One of these modalities is focused ultrasound cavitation, which conveys focused acoustic energy to certain depths in the subcutaneous tissue, and the procedure has been approved by the US Food and Drug Administration [8]. HIFU ablates subcutaneous adipose tissue by generating molecular vibrations that elevate the local tissue temperature and produce rapid cell necrosis [9]. Therefore, combining exercise with HIFU enhances the metabolism of the disrupted fat cells as a source of fuel via the fatty acid oxidation pathway [10]. This is associated with an improvement in the oxidation of fat via an enhanced hormonal response at both the systemic level (i.e. elevated catecholamine and insulin sensitivity) [11] and the local level (i.e. increased skeletal muscle irisin) [12].

Non-invasive ultrasound cavitation can induce similar results in the reduction of fat and body shape as invasive ultrasound liposuction but, it is moderately less expensive, safer, and lower in terms of hazards [13]. Other advantages of non-invasive ultrasound cavitation are the absence of drawbacks and decreases peri-procedural morbidity, such as anesthesia, infection, and scarring, which are common after liposuction surgical procedures [8].

Currently, one of the most important and challenging issues for public health is preventing ectopic fat accumulation and its complications, such as NAFLD. No medications to treat NAFLD are currently available, and the key therapeutic options include weight reduction by exercise and a balanced diet [14]. Therefore, new intervention is needed that can address the underlying risk factors, such as abdominal obesity, and mange fatty liver disease. Ultrasound cavitation is a new, non-invasive alternative to liposuction that has been developed to minimize body fat [15]. The authors believe that focused ultrasound cavitation could enhance and speed up the liver fat reduction as a consequence of abdominal fat reduction. To the best of the authors’ knowledge, there are limited studies in the literature to confirm the effect of focused ultrasound cavitation on abdominal and intrahepatic fat. Therefore, this study aimed to investigate the effect of focused ultrasound cavitation augmented with aerobic exercise on intra-abdominal visceral adipose tissues and liver fats as a response to ablation of subcutaneous adipose tissues in fatty liver patients. The findings of this study can assist both patients with abdominal obesity or fatty liver disease and health care practitioners by providing information about a non-invasive effective treatment for abdominal obesity and liver fat reduction.

## Materials and methods

This parallel-group randomized controlled trial included only 34 patients (male and female) out of the 50 patients who were eligible in this study. The study was conducted at El Sahel Teaching Hospital, Giza, Egypt. Ethical approval was obtained from the Institutional Review Board of the Faculty of Physical Therapy, Cairo University before starting the study No: P.T REC/012/00451. The patients signed an informed consent before enrollment in the study. The preparation and recruitment for the study started in March 2019. This study was registered at ClinicalTrials.gov (NCT04161703, initial release 11/09/2019). The actual study took place between November 2019 and March 2020). All tests were performed in accordance with the Declaration of Helsinki.

The patients included in this randomized controlled trial were aged 30–45 with a body mass index (BMI) 30–40 kg/m2, fatty liver disease according to the liver echogenicity in ultrasound assessment, a liver-to-spleen ratio of less than 1, and a waist of circumference greater than 88 cm for females, and greater than 102 cm for males. The participants were randomly assigned equally to two groups, using focused ultrasound and moderate aerobic exercise in the study group (Group A) and using moderate aerobic exercise only in the control group (Group B).

After the eligible patients were recorded, they were randomly allocated to each group with an allocation ratio of 1:1 by selecting blindly numbers from sealed envelopes, created by a random block randomization technique. In sequential order, the participants were assigned to group A or B. The participants, outcome assessors, and data analyst were blind to the group allocation in the study.

The participants who had the following were excluded: liver diseases such as hepatitis; hepatocellular carcinoma; decompensated liver cirrhosis; uncontrolled diabetes or autoimmune diseases; abdominal hernias with metallic sections; pregnant women; intrauterine devices or a pacemaker; articular prosthesis; a reduced nervous sensibility or with neurological pathologies. Participants who had followed an exercise or weight reduction program in the previous six months were also excluded.

Throughout the study, the participants were instructed to avoid performing other physical exercises and using any products used for weight reduction or that could change the skin appearance (for example, retinoids and vitamin creams). Furthermore, topical steroids were avoided on the treatment site for about two months before the treatment to avoid the impact of topical steroids on the inflammatory reaction to ultrasound cavitation post-treatment and to evaluate any complications that may occur during and after the application of focused ultrasound cavitation.

### Outcome measures

All outcome measures were measured at the beginning of the study and 3 months later by two assessors who were blind to group allocation. Primary outcome measures were abdominal subcutaneous fat volume, abdominal visceral fat volume, and L/S ratio. Secondary outcome measures were body weight, BMI, and waist circumference. A computed tomography device, light speed 16, 120 KV, made by General Electric Medical Systems Co., USA, was used to measure the abdominal subcutaneous, visceral fat size, and L/S ratio for all patients in both groups. A weight and height analog scale (made in China) was used for measuring the body weight and height of all patients and calculating their BMI. Waist circumference was measured at the midway between the lowest rib and the iliac crest.

#### Primary outcome measures

*1- Liver to spleen ratio (L/S ratio) measurement*. A recent study stated that liver attenuation measurement by unenhanced CT scans is the best way of predicting pathological fat content in the liver [16].

Thus, estimation of liver and spleen attenuation in Hounsfield units was measured by abdominal multi-detector CT scans at the level of the T12 to L1 intervertebral space. The liver attenuation was determined by calculating the mean Hounsfield units of 3 regions of interest in the parenchyma of the liver and also of the spleen. The L/S ratio for CT attenuation values is the index for liver fat degree, with an L/S ratio of less than 1 considered to represent fatty liver disease [17].

*2- The abdominal fat volume measurements*. The abdominal fat volume was measured by a new advanced application called "Synapse 3D Lung and Abdomen Analysis". Synapse 3D Lung and Abdomen Analysis is an application developed by FUJIFILM MEDICAL SYSTEMS, U.S.A., Inc. that can perform volume calculation for pulmonary nodes, display low absorption areas, and perform other analysis for lung contrasted and non-contrasted CT volume date. Additionally, the application can calculate the area and volume (3D) of subcutaneous fat and visceral fat using abdominal CT images. The result can be displayed as a graph, and the fat quantity at each slice position can be presented.

### Treatment procedures

To minimize possible nutritional bias, all subjects in both groups followed a weight maintenance diet, which ranged from 1700 kcal to 2200 kcal/day, which was calculated on an individual basis for each subject according to the requirement of each participant and their basal metabolic rate (BMR). The Harris–Benedict equation was used to calculate BMR (BMR = (10 × weight in kg) + (6.25 × height in cm)–(5 × age in years)– 161). The subjects’ energy requirements were calculated by multiplying their BMR by a factor of 1.55 [18]. The diet composition was low fat from 20 to 25%, sufficient in protein from 25 to 30%, and carbohydrates from 50 to 60%. No nutritional supplements were prescribed. A dietitian interviewed the participants in individual counseling sessions. To ensure adherence to the planned diet, the dietitian reviewed the 3-day food diaries on a weekly basis for each participant. The dietitian was blind to the group assignment.

All subjects in both groups followed an aerobic exercise program under direct supervision at a frequency of 3 times/week for 12 weeks, in the form of walking on a treadmill for 30 minutes with moderate intensity (12–14, according to Borg Scale) [19] with 5-minute warm-up and cool-down periods. Each subject in both groups (A & B) was instructed to drink plenty of water during the period of the study, to maintain normal body hydration.

Subjects in group (A), received focused ultrasound cavitation using the device "Mabel 6 DUO. Ultra Cavitation Technology produced by DAEYANG MEDICAL CO., KOREA. HIFU". Ultrasound waves applied to the abdominal region, which extended bilaterally from the line extending from mid-axilla to iliac crest, and above from center of diaphragm to the line extending between the two iliac crests below. The patient was in a comfortable, supine lying position during the treatment session. Ultrasound pulsed waves were delivered with a frequency 40 kHz through a transducer with a diameter of 8.0 cm and a power of 45W. The focused ultrasound session was 30 minutes, once a week for 12 weeks.

### Power analysis of the study

Based on the pilot study of 5 patients for each group, the sample size was calculated according to the independent t-test using the difference in the mean value of subcutaneous fat volume between group A (15255.97 ± 968.004) and group B (14160.278± 244.58) measured pretreatment, with an effect size of 1.552. Assuming (α = 0.05, power of 95%), a sample size of 10 patients per group would be needed [(G) Power 13]. Fifty subjects were recruited to account for estimated dropout rates.

### Statistical analysis

Statistical analysis was conducted using SPSS version 22 (SPSS, Inc., Chicago, IL). Data were screened for normality assumption before final analysis, using the Shapiro-Wilk method which showed that the data were normally distributed and did not violate the parametric assumption. Consequently, the independent t-test was used to compare between groups regarding the baseline characteristics. A 2×2 mixed design MANOVA was used to compare the selected parameters with different tested groups and measuring periods. The F value was calculated using Wilks’ lambda, and when the MANOVA revealed a significant time × group interaction effect (*P* < 0.05), follow‐up univariate ANOVAs were executed. The level of significance was *p* ≤ 0.05.

## Results

**Fig 1:**
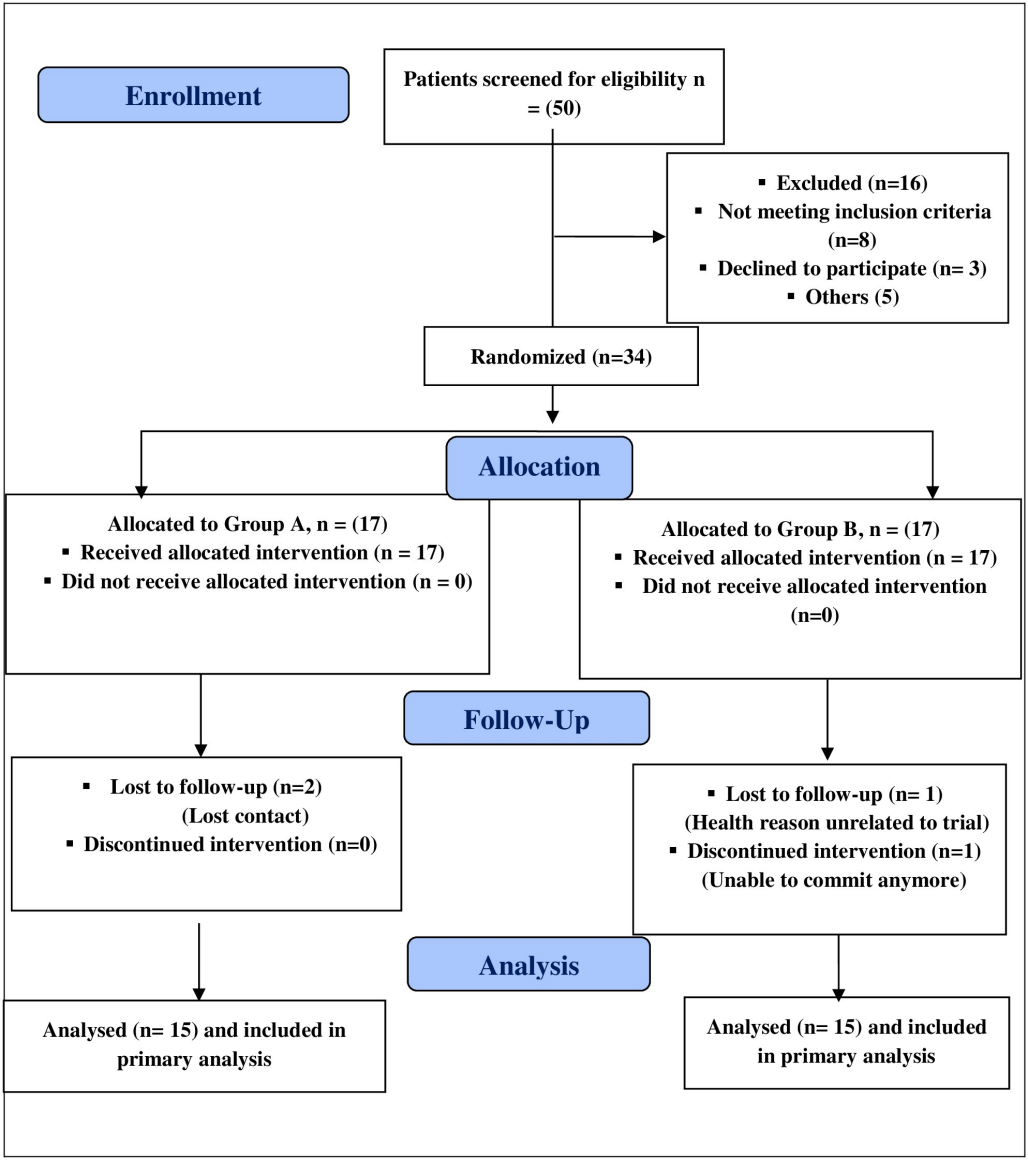
Consort diagram for the study.

Fig 1 indicates the flow of participants from recruitment to follow-up; 50 subjects were assessed for eligibility and 34 subjects were equally randomly assigned to each group, and only 4 subjects were omitted from follow-up. Results include data of 30 participants who completed the study, 15 in each group. Data review revealed no significant variations between the two groups in terms of demographic features and clinical parameters pre-intervention, where (P > 0.05), as demonstrated in Table 1.

**Table 1:** Baseline demographic and clinical characteristics of patients.

Characteristics	Study group	Control group	Mean difference	95% CI	*P* Value
	Mean ± SD	Mean ± SD			
**Age (year)**	35.13±3.5	36.73±3.49	-1.60	(-4.21, 1.01)	0.221
**Height (cm)**	166.8±7.45	168.4±7.7	-1.60	(-7.27, 4.07)	0.568
**Weight (kg)**	103.13±10.32	102.66 ±9.8	0.46	(-7.06, 7.99)	0.090
**BMI (Kg/m**^**2**^**)**	36.98±2.21	36.18 ±2.79	0.80	(-1.08, 2.69)	0.388
**WC (cm)**	119.93±9.87	118.2 ±7.43	1.73	(-4.80, 8.27)	0.591
**SFV (cm**^**3**^**)**	12927.01±2816.9	13073.89 ±2806.4	-146.87	(-2249.95, 1956.19)	0.887
**VFV (cm**^**3**^**)**	3681.79±1039.78	3973.88 ±787.66	-29208	(-982.00, 397.82)	0.393
**Liver/spleen ratio**	0.85±0.09	0.82 ±0.09	0.02	(-0.04, 0.090	0.511

SD = standard deviation; CI = confidence interval; BMI = body mass index; WC = waist circumference; SFV = subcutaneous fat volume; VFV = visceral fat volume; level of significance at *P* ≤ 0.05.

The results showed a statistically significant time × group interaction effect on the combined dependent variables, F (6, 51) = 5.474, *P* < 0.0001, η^2^ = 0.39; Wilks’ Λ = .60. This interaction effect indicates that the difference between groups on a linear combination of the outcome measures is different at post-test than pre-test. However, follow‐up univariate ANOVAs reveal a significant change in waist circumference, F (1,56) = 6.11, *P* < 0. 01, η^2^ = 0.17, and liver/spleen ratio, F (1,56) = 22.93, *P* < 0.0001, η^2^ = 0.23.

**Fig 2:**
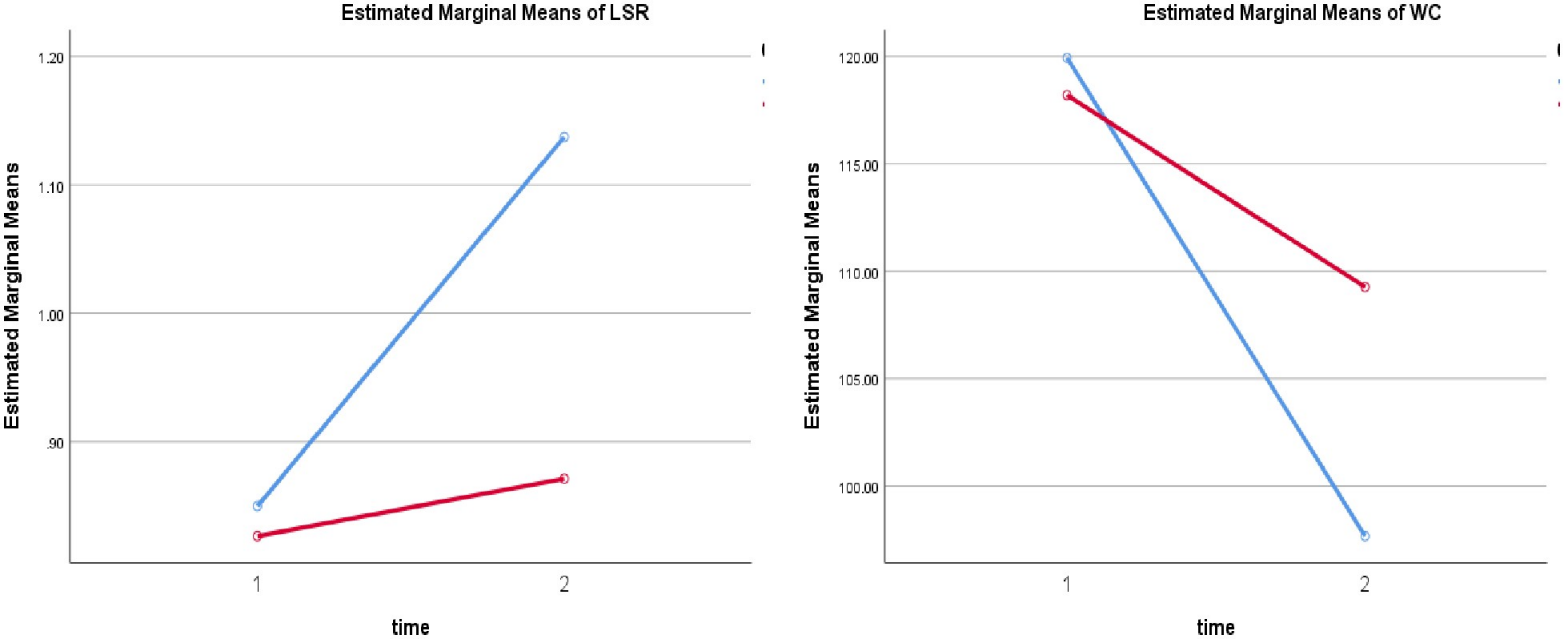
Liver/spleen ratio (LSR) and waist circumference (WC) in the two groups pre- & post-intervention.

After treatment, there was a significant improvement in liver/spleen ratio of the study group compared to the control group (*P* < 0.0001), study group 22.27 [CI 95%: 19.20, 25.32, *P* <0.0001]; control group 8.94 [CI 95%: 5.87, 11.99, *P* <0.0001] and a significant decrease in waist circumference of the study group relative to the control group (*P* < 0.0001), study group -0.28 [CI 95%: -0.33, -0.23, *P* = 0.001]; with no significant change compared to baseline in the control group -0.05 [CI 95%: -0.09, 0.004, *P* = 0.07] as shown in Tables 1 and 2. The results are illustrated in Fig 2.

**Table 2:** Weight, body mass index, waist circumference, subcutaneous fat volume, visceral fat volume, and Liver/spleen ratio post-intervention.

Characteristics	Study group	Control group	Mean difference	95% CI	*P* Value
	Mean ± SD	Mean ± SD			
**Weight (kg)**	83.73 ±7.77	89.06±8.89	5.33	(-11.58, 0.91)	0.091
**BMI (Kg/m**^**2**^**)**	30.08 ±2.59	31.41±3.22	-1.33	(-3.52, 0.85)	0.222
**WC (cm)**	97.66 ±7.5	109.26±5.44	-11.60	(-16.50, -6.69)	0.0001*
**SFV (cm**^**3**^**)**	9581.47±2901.94	11619.75 ±3076.33	-2038.27	(-4275.02, 198.46)	0.072
**VFV (cm**^**3**^**)**	3230.30±912.16	3824.62±745.74	-594.32	(1217.47, 28.82)	0.061
**Liver/spleen ratio**	1.13 ±0.15	0.87±0.11	0.26	(0.165, 0.36)	0.0001*

SD = standard deviation; CI = confidence interval; BMI = body mass index; WC = waist circumference; SFV = subcutaneous fat volume; VFV = visceral fat volume;

* = Significant; level of significance at *P* ≤ 0.05.

Concerning, body weight, BMI, WC, abdominal subcutaneous fat, and visceral fat, within-group analysis revealed a significant difference in the two groups after 12 weeks of the intervention relative to baseline where *(P < 0*.*0001)*, with greater improvements in the intervention group. While regarding the liver/spleen ratio, only the intervention group displayed a substantial difference, where *(P < 0*.*0001)*. The results are illustrated in Tables 3 and 4.

**Table 3:** Weight, body mass index, waist circumference, subcutaneous fat volume, visceral fat volume, and Liver/spleen ratio pre- and post-intervention for study group.

Characteristics	Pre	Post	Mean difference	95% CI	*P* Value
	Mean ± SD	Mean ± SD			
**Weight (kg)**	103.13±10.32	83.73 ±7.77	19.40	(16.85, 21.94)	<0.0001*
**BMI (Kg/m**^**2**^**)**	36.98±2.21	30.08 ±2.59	6.90	(6.16, 7.65)	<0.0001*
**WC (cm)**	119.93±9.87	97.66 ±7.5	22.27	(19.20, 25.32)	<0.0001*
**SFV (cm**^**3**^**)**	12927.01±2816.9	9581.47±2901.94	3345.53	(2910.24, 3780.83)	<0.0001*
**VFV (cm**^**3**^**)**	3681.79±1039.78	3230.30±912.16	451.49	(368.06, 534.91)	<0.0001*
**Liver/spleen ratio**	0.85±0.09	1.13 ±0.15	-0.28	(-0.33, -0.23)	0.001*

SD = standard deviation; CI = confidence interval; BMI = body mass index; WC = waist circumference; SFV = subcutaneous fat volume; VFV = visceral fat volume;

* = Significant; level of significance at *P* ≤ 0.05.

**Table 4:** Weight, body mass index, waist circumference, subcutaneous fat volume, visceral fat volume, and Liver/spleen ratio pre- and post-intervention for control group.

Characteristics	Pre	Post	Mean difference	95% CI	*P* Value
	Mean ± SD	Mean ± SD			
**Weight (kg)**	102.66 ±9.8	89.06±8.89	13.60	(11.05, 16.14)	<0.0001*
**BMI (Kg/m**^**2**^**)**	36.18 ±2.79	31.41±3.22	4.76	(4.02, 5.51)	<0.0001*
**WC (cm)**	118.2 ±7.43	109.26±5.44	8.94	(5.87, 11.99)	<0.0001*
**SFV (cm**^**3**^**)**	13073.89 ±2806.4	11619.75 ±3076.33	1454.13	(1018.84, 1889.43)	<0.0001*
**VFV (cm**^**3**^**)**	3973.88 ±787.66	3824.62±745.74	149.25	(65.82, 232.67)	0.001*
**Liver/spleen ratio**	0.82 ±0.09	0.87±0.11	-0.05	(-0.09, 0.004)	0.072

SD = standard deviation; CI = confidence interval; BMI = body mass index; WC = waist circumference; SFV = subcutaneous fat volume; VFV = visceral fat volume;

* = Significant; level of significance at *P* ≤ 0.05.

## Discussion

Currently, NAFLD is the most widely recognized type of chronic liver disease, and its prevalence has increased in parallel with the increase in obesity prevalence [20]. Therefore, this study aimed to detect the effect of focused ultrasound augmented with aerobic exercise as a noninvasive procedure for abdominal and liver fat reduction in NAFLD patients. This clinical study demonstrated that focused ultrasound cavitation, augmented with aerobic exercise, is effective in decreasing abdominal contouring and intrahepatic fats. It significantly reduced excess adipose tissue in the abdomen, as reflected by the decrease in waist circumference; abdominal subcutaneous and visceral fat volume; and increased liver-to-spleen ratio of the participants with a higher percentage of improvement than the control group.

Several previous studies reported a reduction in waist circumference after HIFU treatments [21–23], and the results of this study are consistent with Baldi et al. [24], who demonstrated that focused ultrasound cavitation results in decrease subcutaneous fat thickness in the treated area, and was a safe and effective method for body contouring. The recorded reduction in waist circumference was nearly 6.2 cm after 2 months of treatment. Moreover, the results of the current study were consistent with the results of Fatemi et al. [25], who found an average reduction of about 4.7 cm in waist circumference post-application of HIFU to the abdomen and flanks after 3 months. The authors reported that no visible damaged cells appeared after the treatment. Furthermore, Lee et al. [26] investigated the immediate tissue reactions to focused HIFU via ultrasound images, and reported round-to-oval ablative thermal injury zones in the subcutaneous fat layers of abdomen and thigh from a human cadaver.

Recently, in a study by Gold et al. [27], three consecutive ultrasound treatments led to significant fat decrease measured by ultrasound imaging of treated flanks, in comparison to the untreated controls. Likewise, in a study by Moreno-Moraga et al. [21], 30 healthy patients underwent three HIFU treatment sessions at one-month intervals. The participants were assessed one month after the end of treatment. There was a significant decrease of subcutaneous fat thickness in the abdomen and other treated areas. During the treatment procedure, cholesterol levels did not change and triglyceride levels increased slightly. Additionally, one month after the first session, liver ultrasound assessment was normal in all patients, indicating that triglycerides released and did not cause fat accumulation in the liver.

In contrast to these results, Shek et al. [28] observed an abdominal circumference increase by 2.03 cm after three sessions of focused ultrasound treatment. This difference in their results may be due to body size diversity and measurement bias because of abdominal skin laxity after lipolysis, as well as a lack of monitoring of the participants’ diets. Furthermore, Moravvej et al. [29] found a reduction of 1.8 cm in circumference during the ultrasound session, but more episodes of focused ultrasound did not lead to a greater circumference reduction. This could be attributed to a lack of dietary change during the treatment period, in addition to other limitations such as the lack of a randomized control group.

The underlying mechanism of focused ultrasound cavitation is that ultrasound energy prompts therapeutic regeneration as well as destruction of dermal parts and subcutaneous fat tissues through coagulation necrosis and acoustic cavitation in focused tissues [30]. In cellular and subcellular components, pressure-driven cavitation bubbles are formed by ultrasound irradiation through a quick expansion of gaseous nuclei [31]. As the acoustic cavitation breaks down, excessive amounts of energy are moved to surrounding structures [31]. Also, ultrasound waves vibrate composite molecules, which create frictional heat and coagulation necrosis [26]. This cavitation leads to lysis of fat cells while maintaining the surrounding vasculature [32]. At that point, the necrotic cell triggers a wound-healing response, so the macrophages and other cells migrate to the area to clear the destroyed adipocytes’ cellular debris and lipids, leading to a decrease of local fat stores. Consequently, to the wound-healing reaction, fibroblasts and inflammatory cells attracted to the area accompanied by collagen denaturation by the heat lead to shortening and thickening of collagen fibers and contraction [33].

Lifestyle modification by diet or exercise proved to be an effective management or treatment for NAFLD [34]. Although the underlying mechanisms of exercise in liver fat reduction are still unknown, it was evident from the available literature that physical exercise has been shown to reduce liver fat content through improvements in liver fatty acid metabolism, liver mitochondrial function, insulin resistance, and inflammatory cascades activation [35].

A possible explanation of the result of this study is that both the potential lysis of fat cells induced by focused ultrasound cavitation and the greater activation of fatty acid metabolism by exercise are beyond the improvement observed in abdominal and liver fat reduction. In this study, focused ultrasound cavitation augmented with aerobic exercise procedure provides better effective treatment in decreasing abdominal contouring and liver fat. No or minimal discomfort was reported during and after the treatment procedure, and no adverse events occurred during the course of the study.

## Conclusion

Focused ultrasound cavitation augmented with aerobic exercise can be an effective non-invasive procedure for abdominal and liver fat reduction in non-alcoholic fatty liver patients. Focused ultrasound cavitation was applicable and could be a future direction to speed up the liver fat reduction.

## Strength and limitations

The study has several strengths, including its novelty and using CT scans as an objective measurement of subcutaneous and visceral fat, and the best way for assessing the liver fat. Validity of the study’s finding was enhanced through the aspects of study design including randomization, the assessors of the outcome measures were blind to the group allocation, and direct monitoring of the intervention by the investigator. The possible nutritional bias in the study was minimized by providing a weight maintenance diet under direct supervision of a dietitian.

The study’s limitations were the absence of a group treated with focused ultrasound cavitation alone as it is important to enhance lipolysis via diet restriction and/or exercise, and the absence of follow-up assessments meant we could not analyze the long-term effects of the intervention. Another limitation may be the relatively small number of participants who completed the study, so future research needs to be conducted on a large sample and in different settings to confirm the generalizability of the findings.

## Supporting information

S1 Appendix Raw data of the study. (DOC)

S2 Appendix Study protocol. (DOC)

S1 Checklist CONSORT 2010 checklist of information to include when reporting a randomised trial*. (DOC)
