# Supplementary material for: Effect of focused ultrasound cavitation augmented with aerobic exercise on abdominal and intrahepatic fat in patients with non-alcoholic fatty liver disease: A randomized controlled trial
Source: PLoS One. 2021 Apr 28;16(4):e0250337. doi: 10.1371/journal.pone.0250337 (PMC8081198; doi:10.1371/journal.pone.0250337)
Supplement: S1 Appendix — (DOC) [file pone.0250337.s001.doc]

Table 1 Measurements of Study Group (Cavitation and exercise)

| **N** | **Male**  **/**  **Fem-ale** | **Age**  **Years** | **Height**  **Cm** | **Anthropometric measurements** | | | | | | | **C.T measurements** | | | | | |
| --- | --- | --- | --- | --- | --- | --- | --- | --- | --- | --- | --- | --- | --- | --- | --- | --- |
|  |  |  |  | **Weight**  **Kg** | | **BMI**  **kg/m²** | |  | **Waist**  **Circumference cm** | | **Subcutaneous fat**  **(cm³)** | | **Visceral fat**  **(cm³)** | | **Liver/ Spleen**  **Ratio** | |
|  |  |  |  | **Before** | **After** | **Before** | **After** |  | **Before** | **After** | **Before** | **After** | **Before** | **After** | **Before** | **After** |
| **1** | **F** | **36** | **160** | **102** | **83** | **39.8** | **32.4** |  | **127** | **107** | **14863.84** | **11730.88** | **3267.86** | **2931.11** | **0.72** | **1.00** |
| **2** | **F** | **31** | **165** | **105** | **94** | **38.5** | **34.5** |  | **122** | **109** | **16667.4** | **14396.74** | **2540.3** | **2351** | **0.99** | **1.25** |
| **3** | **F** | **30** | **156** | **97** | **84** | **39.8** | **34.5** |  | **110** | **96** | **14785.65** | **12570.80** | **2496.73** | **2218.66** | **0.97** | **1.27** |
| **4** | **F** | **32** | **165** | **94** | **79** | **34.5** | **29.0** |  | **107** | **94** | **10097.53** | **6160.54** | **2084.32** | **1780.8** | **0.99** | **1.44** |
| **5** | **F** | **35** | **162** | **105** | **87** | **40.0** | **33.1** |  | **129** | **108** | **15765.82** | **12339.74** | **4296.87** | **4074.38** | **0.77** | **1.10** |
| **6** | **F** | **31** | **167** | **98** | **77** | **35.1** | **27.6** |  | **109** | **87** | **16234.55** | **12663.19** | **3526.3** | **3083.9** | **0.81** | **1.42** |
| **7** | **F** | **38** | **159** | **84** | **69** | **33.2** | **27.2** |  | **105** | **87** | **10537.26** | **7552.33** | **3245.36** | **2930.44** | **0.81** | **1.08** |
| **8** | **F** | **33** | **163** | **99.5** | **78** | **37.4** | **29.3** |  | **113** | **90** | **14197.16** | **11036.73** | **2347.58** | **1883.70** | **0.92** | **1.17** |
| **9** | **F** | **39** | **169** | **111.5** | **93** | **39.0** | **32.5** |  | **124** | **103** | **17346.83** | **12548.13** | **4528.86** | **3997.24** | **0.71** | **0.98** |
| **10** | **F** | **38** | **158** | **88** | **72** | **35.2** | **28.8** |  | **110** | **92** | **11364.30** | **7478.92** | **2984.26** | **2663.17** | **0.89** | **1.05** |
| **11** | **M** | **38** | **173** | **103** | **81** | **34.4** | **27.0** |  | **122** | **91** | **9380.56** | **6681.13** | **4796.3** | **4371.89** | **0.87** | **1.06** |
| **12** | **M** | **36** | **177** | **113** | **88** | **36.0** | **28.0** |  | **129** | **98** | **10837.52** | **7375.930** | **4963.27** | **4378.63** | **0.78** | **0.98** |
| **13** | **M** | **31** | **180** | **120** | **94** | **37.0** | **29.0** |  | **134** | **102** | **11179.54** | **7632.760** | **4570.27** | **3868.81** | **0.91** | **1.21** |
| **14** | **M** | **39** | **176** | **119** | **91** | **38.4** | **29.3** |  | **131** | **104** | **10183.89** | **6987.130** | **4761.85** | **3863.71** | **0.84** | **1.11** |
| **15** | **M** | **40** | **172** | **108** | **86** | **36.5** | **29.0** |  | **127** | **97** | **10463.32** | **6567.180** | **4816.73** | **4057.13** | **0.77** | **0.94** |

Table 2 Measurements of Control Group

| **N** | **Male**  **/**  **Female** | **Age**  **Years** | **Height**  **Cm** | **Anthropometric measurements** | | | | | | | | **C.T measurements** | | | | | |
| --- | --- | --- | --- | --- | --- | --- | --- | --- | --- | --- | --- | --- | --- | --- | --- | --- | --- |
|  |  |  |  | **Weight**  **Kg** | | | **BMI**  **kg/m²** | |  | **Waist**  **Circumference cm** | | **Subcutaneous Fat**  **(cm³)** | | **Visceral Fat**  **(cm³)** | | **Liver/ Spleen**  **Ratio** | |
|  |  |  |  | **Before** | | **After** | **Before** | **After** |  | **Before** | **After** | **Before** | **After** | **Before** | **After** | **Before** | **After** |
| **1** | **F** | **31** | **165** | **106** | | **100** | **38.9** | **36.7** |  | **113** | **114** | **15398.29** | **16471.14** | **3298.05** | **3239.71** | **0.93** | **0.92** |
| **2** | **F** | **37** | **163** | **91** | | **85** | **34.2** | **31.9** |  | **115** | **110** | **13847.52** | **13284.15** | **4374.86** | **4315.14** | **0.90** | **0.91** |
| **3** | **F** | **44** | **165** | **109** | | **100** | **40.0** | **36.7** |  | **117** | **112** | **14176.54** | **13297.86** | **3585.37** | **3495.69** | **0.98** | **1.03** |
| **4** | **F** | **33** | **159** | **89** | | **74** | **35.2** | **29.2** |  | **112** | **105** | **14945.37** | **13070.80** | **3379.77** | **3310.76** | **0.78** | **0.81** |
| **5** | **F** | **35** | **161** | **98** | | **85** | **37.8** | **32.7** |  | **110** | **104** | **15167.77** | **14087.36** | **2386.57** | **2326.94** | **0.76** | **0.80** |
| **5** | **F** | **39** | **167** | **106** | | **89** | **38.0** | **31.9** |  | **116** | **107** | **13986.63** | **11874.04** | **3167.41** | **3008.12** | **0.87** | **1.11** |
| **6** | **F** | **38** | **170** | **93** | | **82** | **32.1** | **28.3** |  | **113** | **106** | **14378.92** | **12006.13** | **3941.03** | **3879.57** | **0.81** | **0.82** |
| **7** | **F** | **41** | **156** | **92** | **77** | | **37.8** | **31.6** |  | **112** | **104** | **14411.78** | **12397.11** | **4364.47** | **4038.57** | **0.74** | **0.87** |
| **9** | **F** | **36** | **167** | **109** | **97** | | **39.0** | **34.7** |  | **125** | **116** | **16345.83** | **15236.28** | **3174.94** | **2994.34** | **0.95** | **0.97** |
| **10** | **F** | **39** | **175** | **111** | **90** | | **36.2** | **29.3** |  | **122** | **111** | **16178.64** | **13738.37** | **3962.86** | **3785.97** | **0.78** | **0.79** |
| **11** | **M** | **37** | **165** | **101** | **92** | | **37.0** | **33.7** |  | **118** | **111** | **8488.59** | **7661.19** | **4636.30** | **4393.89** | **0.97** | **0.98** |
| **12** | **M** | **35** | **179** | **107** | **91** | | **33.3** | **28.4** |  | **125** | **110** | **9826.84** | **7313.27** | **4885.67** | **4694.58** | **0.71** | **0.73** |
| **13** | **M** | **37** | **178** | **96** | **80** | | **30.2** | **25.2** |  | **112** | **99** | **8226.63** | **7089.43** | **4462.81** | **4315.52** | **0.80** | **0.81** |
| **14** | **M** | **31** | **182** | **126** | **105** | | **38.0** | **31.6** |  | **137** | **121** | **10974.54** | **9132.36** | **5070.77** | **4868.11** | **0.69** | **0.75** |
| **15** | **M** | **38** | **174** | **106** | **89** | | **35.0** | **29.3** |  | **126** | **109** | **9754.47** | **7636.83** | **4917.32** | **4702.52** | **0.73** | **0.77** |
