# Supplementary material for: Effect of focused ultrasound cavitation augmented with aerobic exercise on abdominal and intrahepatic fat in patients with non-alcoholic fatty liver disease: A randomized controlled trial
Source: PLoS One. 2021 Apr 28;16(4):e0250337. doi: 10.1371/journal.pone.0250337 (PMC8081198; doi:10.1371/journal.pone.0250337)
Supplement: S2 Appendix — (DOC) [file pone.0250337.s002.doc]

| **FACULTY OF PHYSICAL THERAPY**  **CAIRO UNIVERSITY**  **ETHICAL REVIEW** |
| --- |

**Title : Response of Abdominal Fats to Focused Ultrasound in Non-Alcoholic Fatty Liver Patients. Study Protocol**

**Introduction**

In the past decades, the prevalence of overweight and obesity has increased rapidly worldwide. Obesity is a major risk factor for several chronic diseases such as type 2 diabetes mellitus (T2DM), cardiovascular disease and some kinds of cancer **(Yunjuan et al., 2013).**

Obesity is not only a serious health and economic burden, but also predisposes a person to a variety of metabolic diseases. Metabolic syndrome can be characterized by a group of metabolic risk factors that includes central obesity, insulin resistance, dyslipidemia, increased blood pressure, and nonalcoholic fatty liver disease (NAFLD) **(Akagiri et al., 2008).**

Non-alcoholic fatty liver disease (NAFLD) is defined as the excessive and abnormal intracellular accumulation of lipid in the liver, primarily in the form of triglycerides, in the absence of alcohol consumption and other causes of chronic liver disease such as viral hepatitis or drugs. NAFLD is a broad spectrum of disease, ranging from simple steatosis to non-alcoholic steatohepatitis (NASH) **(Lee, 2017).**

The high prevalence of Non-alcoholic fatty liver disease (NAFLD) is probably due to the contemporary epidemics of obesity, unhealthy dietary pattern, and sedentary lifestyle. NAFLD patients are at increased risk of cardiovascular and liver related mortality. The cornerstone of any treatment regimen for patients with NAFLD is lifestyle modification focused on weight loss, exercise, and improving insulin sensitivity **(Al-Dayyat et al., 2018****)**

Most people with [nonalcoholic fatty liver disease](https://www.sciencedirect.com/topics/medicine-and-dentistry/non-alcoholic-fatty-liver-disease) (NAFLD) are obese, with a reported prevalence as high as 80% in obese patients and only 16% in individuals with a normal Body Mass Index (BMI) and without metabolic risk factors (**Kwak et al., 2017) and (Milić et al., 2014).**

It has been reported that about 30% of the obese male and 40% of the obese female have NAFLD. Nevertheless, if we consider only the obese patients with Insulin Resistance (IR), and/or T2DM, the reported prevalence is increased and ranges from 30% to 100%. The major pathogenetic determinant of the NAFLD occurrence is systemic IR that is independent of weight, percentage of body fat and visceral fat mass **(Abenavoli et al., 2016).**

Obesity-related health risk is greatest in those with an abdominal obesity phenotype. Specifically, excess accumulation of visceral fat within the abdomen is strongly and independently associated with morbidity and mortality more closely than with the BMI itself or the amount of subcutaneous fat **(Janiszewski et al., 2007).**

The association of central adiposity to poor health is related to Visceral Adipose Tissue (VAT) accumulation and associated hyperinsulinemia. Increased VAT is associated with elevated free fatty acids, which impair hepatic insulin clearance, resulting in hyperinsulinemia, increased gluconeogenesis, and elevation of very-low-density lipoprotein secretion. Very-low-density lipoprotein secretion and insulin are independent predictors of myocardial infarction **(Riechman et al., 2002).**

Both fatty liver and abdominal visceral adipose tissue (VAT) are important risk factors for the development of cardio metabolic complications due to obesity. Epidemiological studies indicate that higher levels of VAT or fatty liver are associated with insulin resistance, metabolic syndrome, dyslipidemia, hypertension, and diabetes. Moreover, because of the anatomic blood circulation between VAT and the liver, free fatty acids and inflammatory adipokines that are produced by VAT can be released into the portal vein and directly transported to the liver, causing fatty liver disease. These observations have led to a hypothesis that fatty liver may be another important characteristic of fat distribution that is associated with different metabolic risk profiles **(Lonardo et al., 2015) and (Liu et al., 2011).**

The risks, financial costs and lengthy downtime associated with surgical procedures for fat reduction have led to the development of a number of non-invasive techniques **(Kennedy et al., 2015).**

High-intensity-focused ultrasound (HIFU), nonsurgical method of body contouring that involves the use of high-HIFU for thermo coagulation-mediated fat removal. The HIFU uses the same energy source as a diagnostic ultrasound and relies on the same principles. Ultrasound propagates harmlessly through tissue, but if the ultrasound beam carries sufficient energy and is focused, it can cause a local rise in temperature to cause necrosis of tissue **(Kennedy et al., 2003).**

The predictable and reproducible effects of HIFU have made it a promising tool for treating a variety of diseases and medical conditions. Current therapeutic applications of HIFU in human medicine include the treatment of cancer and solid tumors, leiomyomas and atrial fibrillation **(Gadsden et al., 2011).**

High-intensity-focused ultrasound (HIFU) can be focused at specific depths in subcutaneous adipose tissue, preventing exposure and damage to tissues outside of the focal zone. The intensity is brought to a sharp focus in the subcutaneous fat to destroy adipocytes by causing molecular vibrations that increase the temperature of local tissue and induce rapid cell necrosis. Lesion healing and lipid resorption follow normal healing processes with the gradual metabolism of free lipids **(Jewell et al., 2012) and (TerHaar et al., 2007).**

So, the aim of the present study was to investigate the response of abdominal fat to focused ultrasound in fatty liver patients.

**Statement of the problem: -**

The problem of this study was stated in the following question:

Will there a significant effect of Focused Ultrasound on the abdominal fats in fatty liver patients?

**Purpose of the study: -**

The purposes of the study will be to study the effect of Focused Ultrasound on body weight, BMI, waist circumference, abdominal subcutaneous fat, visceral fats and liver to spleen ratio in nonalcoholic fatty liver patients with mild to moderate obesity.

**Significance of the study: -**

Obesity, a major health problem worldwide, is defined as a fat storage disease. The excessive accumulation of visceral (intra-abdominal) adipose tissue correlates with metabolic syndrome features in obese and overweight individuals. According to the overflow hypothesis, increased energy intake could primarily promote the filling of the visceral fat compartment that will further contribute to hepatic and peripheral insulin resistance. The subcutaneous fat (sc) fat appears to play the role of a metabolic sink, buffering dietary fat to limit their deposition in other organs. The adipose tissue expandability hypothesis infers, however, that for some obese individuals, subcutaneous (sc) adipose tissue may reach its maximal storage capacity, the excess of lipids then being reoriented toward other tissues **(Alligier et al., 2013).**

Obesity increases the risk of developing a variety of pathological conditions, including insulin resistance, type 2 diabetes, dyslipidemia, hypertension and non-alcoholic fatty liver disease (NAFLD). NAFLD is currently the most common form of chronic liver disease, and its incidence has increased in parallel to the rise in the incidence of obesity. More than two-thirds of patients with NAFLD are obese. In the obese state, pro-inflammatory and anti-inflammatory factors secreted by inflamed adipose tissue are also associated with NAFLD **(Jung and Choi, 2014).**

Body fat distribution has been shown to pose a greater health risk than overall body fat, and among the different types of obesity based on specific categories of body fat distribution, abdominal obesity has been reported to pose the greatest risk. Visceral abdominal obesity is viewed as the more clinically important type of abdominal obesity. Furthermore, by causing physical pressure, the accumulation of heavy visceral adipose tissue (VAT) can interrupt blood flow to abdominal organs and decrease organ function (e.g., liver). As such, VAT can be even more deleterious than subcutaneous adipose tissue (SAT) (**Kim et al., 2016).**

It has been hypothesized that enlargement of subcutaneous adipocytes is a sign of reduced adipogenic capacity of subcutaneous adipose tissue (SAT), which in turn leads to increased accumulation of fat in visceral adipose tissue (VAT), skeletal muscle, and liver and a subsequent worsening of insulin action and glucose tolerance **(Koska et al., 2008).**

Current Food and Drug Administration (FDA)-approved methods of treating localized, central abdominal adiposity include cryolipolysis, high-intensity focused ultrasound, and low-level laser therapy, relying on the cooling, heating, and physiologic modification of subcutaneous adipose tissue to achieve significant fat reduction **(Friedmann et al., 2014).**

Ultrasound energy induces therapeutic regeneration and/or destruction of dermal components and subcutaneous fat tissues via acoustic cavitation and coagulation necrosis in targeted tissues. Ultrasonic irradiation of cells forms pressure-driven cavitation bubbles in cellular and subcellular structures by rapid expansion of gaseous nuclei. As the acoustic cavitation collapses, extremely high amounts of energy are transferred to surrounding structures. Additionally, ultrasound waves vibrate composite molecules to produce frictional heat and histologic changes of coagulation necrosis **(Sugun Lee et al., 2017).** Most of the cellular debris and lipid is removed within 12 weeks of the procedure, with 95%removed within 18 weeks. This removal process is not associated with any significant impact on plasma lipid levels **(Stephan et al., 2010).**

Focused ultrasound waves used to remove fat cells permanently. Once removed, these cells will not re grow. Ultrasound waves painlessly disrupt the walls of cells containing fat found below the skin within the subcutaneous fat layer, while other tissues such as blood vessels and nerves are unharmed **(Moreno et al., 2007)**.

To the best of our knowledge, still no studies confirm the effect of Focused ultrasound waves to decrease intra-abdominal visceral adipose tissues and liver fats in response to subcutaneous adipose tissues reduction.

This study is a trial to discover the effect of Focused Ultrasound on abdominal fat size in fatty liver patients.

**Hypothesis**: -

There will be no effect of Focused Ultrasound on body weight, BMI, waist circumference, abdominal subcutaneous fat, visceral fats and liver to spleen ratio in fatty liver patients with mild to moderate obesity.

**Materials and Methods**

**I) Patients**

Fifty patients with nonalcoholic fatty liver disease in both sex will be recruited to account for the dropout rates. This study will be conducted at the physical therapy department of EL Sahel Teaching Hospital. They will be assigned randomly into two equal groups: the study group (A) and the controlled group (B).

**Group A:** patients suffering from moderate nonalcoholic fatty liver with mild to moderate obesity will receive High Intensity Focused Ultrasound, diet maintenance and exercises.

**Group B:** patients suffering from moderate nonalcoholic fatty liver with mild to moderate obesity will receive exercises and diet maintenance only.

**Power analysis of the study:**

Based on the pilot study of 5 patients for each group, the sample size was calculated according to the difference in the mean value of subcutaneous fat volume between group A (15255.97 ± 968.004) and group B (14160.278± 244.58) measured pretreatment, with an effect size of 1.552. Assuming α=0.05, power of 90%, so a sample size of 10 patients per group would be needed [(G) Power 13]. Fifty subjects will be recruited to account for the dropout rates.

**Inclusion criteria**

Subjects who participated in this study had the following criteria:

- Moderate nonalcoholic fatty liver.
- Liver to Spleen ratio (LSR) < 1.
- Body mass index (BMI) ranged from 30 to 40 kg/m².
- Waist circumference ≥ 102 cm for men and ≥ 88 cm for women.
- Waist to hip ratio (WHR) ˃ 0.95 for men and ˃ 0.8 for women.
- Sedentary (exercise < 2 times/week).

**Exclusion criteria:**

Subjects had been excluded if they have any of the following:

- Neoplastic or autoimmune diseases.
- History of liver surgery or viral hepatitis.
- Cardiac disorders, osteoporosis, phlebitis and thrombophlebitis.
- Scarring, hernias or skin diseases in the abdominal area.
- Metallic sections, articular prosthesis or a pacemaker.
- Pregnancy or intrauterine devices.
- Any participant underwent other slimming or aesthetical procedures.
- Patients with a reduced nervous sensibility or neurological pathologies.

**Ethical consideration:**

All patients will be informed about the Purpose, nature and potential risks of the study before participating in it. They will sign the consent form and Personal data will be recorded in the first meeting before starting the study

**II) Instrumentation**

Instrumentation in this study will be used for two purposes: -

**(a) Instrumentation for evaluation: -**

1. **Tape measurement:**

Tape measurements will be used to measure the waist and hip circumference for all patients to calculate their waist hip ratio (WHR).

1. **Weight - Height Scale:**

Weight and height analog scale, made in china (**will be** used for measuring the body weight and height of all patients and calculating their Body Mass Index (BMI).

1. **Computed Tomography (CT) device:**

A Computed Tomography (CT) device, light speed16, 120 KV, made by General Electric Medical Systems Co., USA(**Figure 1)** will be used to measure the abdominal subcutaneous, visceral fat volume and assessment of liver fat degree.

**
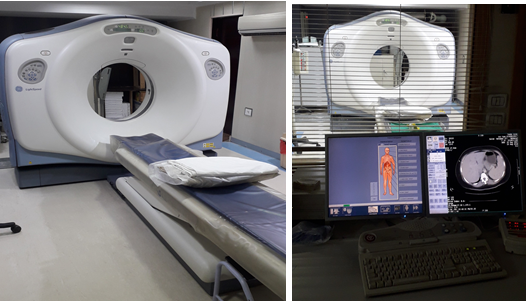
**

**Figure (1):** Computed Tomography

**(b) Instrumentation for treatment:**

**Focused Ultrasound device:**

Focused ultrasound will be applied by The Mabel6 DUO Ultra Cavitation Technology system produced by DAEYANG MEDICAL CO., KOREA. It is an aesthetic multifunction body contouring device which provides Focused Ultrasound Cavitation for fat removal and Multipolar Radio Frequency (RF) System for skin firming. **Multifunction Mabel 6 Duo system applies four kinds of different treatment heads which are small tripolar RF for face, big tripolar & six polar RF for** body skin tightening **and Ultrasound-Cavitation for fat burning** which used in this study (fig 2) with the following specifications:

- Model name: MABEL 6 DUO.
- Power Input: AC100/240V, 50/60Hz.
- Max. Power Consumption: 60 W.
- Output Frequency: 40 KHz.
- Hand probe Diameter: 8.0 cm.
- Output Power: 45W.
- Dimensions: 48(w), 57(D), 95(H) cm.
- Weight: 20 kg.
- Treatment Time: 30 min max.
- Safety Device: Zero Start.


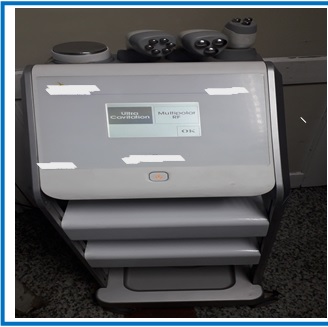


**Figure (2):** Mabel 6 DUO Focused Ultrasound Cavitation

**III) Procedures**

1. **Evaluative procedures:**

The evaluative procedures will be done for all patients in the two groups before starting the program and after 12 weeks of treatment.

- 1. **History taking:**

All data and information of each subject participated in this study was recorded. Detailed medical history was taken.

- 1. **Height, Weight and body mass index (BMI) Measurement:**
- Height of each participant will be measured only before starting the study.
- Body weight of each subject in both groups was measured before and after the program of treatment.
- The body mass index (BMI) of each subject was calculated by dividing the body weight (in kg) by the square of body height (in meters)
- Body mass index (BMI) was taken before starting the study and after the end of treatment.
  1. **Waist circumference (WC) measurements:**
- Waist circumference (WC) will be measured at the midway between the lowest rib and the iliac crest.
- The measurements were taken by Tape measurement for all subjects in both groups (A & B), while the subject in the standing position, with light clothes and at the end of expiration.
  1. **Estimation of the subject's energy requirements (Diet)**

All subjects in both groups will follow a weight maintenance diet**,** which ranged from 1700 kcal to 2200 kcal/day, which will be calculated in an individual basis for each subject according to the requirement of each participant and their BMR, Harris–Benedict equation will be used to calculate BMR, “ BMR=(10×weight in kg) +(6.25×height in cm)–(5×age in years)–161”, The subjects’ energy requirements will be calculated by multiplying BMR and a factor of 1.55. **(Frankenfield et al)**

- 1. **Computed Tomography (CT) Examination**

This examination will be applied for all subjects in both groups (A & B) before starting and after stopping of the study (after 12 weeks).

1. **Measurement of abdominal subcutaneous and visceral fat:**

The abdominal subcutaneous and visceral fat volume will be measured by abdomen computerized tomography image (CT) using an advanced software application called **"Synapse 3D Lung and Abdomen Analysis".**

- ***Description of the application***

"Synapse 3D Lung and Abdomen Analysis" is medical imaging software developed by "FUJIFILM MEDICAL SYSTEMS, U.S.A., Inc." for analyzing the images acquired from CT.

Device Trade Name: Synapse 3D Lung and Abdomen Analysis.

Device Common Name: Medical Image Processing and Analysis Software.

- ***Indications for Use:***
- Using non-contrasted and contrasted enhanced computed tomography images of the lung to perform boundary detection, volume calculation for pulmonary nodes and other analysis in the lung based on the location specified by the user and display low absorption areas.
- Using non-contrasted abdominal CT images for calculating subcutaneous fat and visceral fat areas in 2D and both volumes in 3D. The result can be displayed as a graph, and the fat quantity at each slice position can be presented.

1. **Assessment of liver fat degree:**

The Computed Tomography (CT) assessment of liver fat will be made by measuring the difference in attenuation between the liver and spleen in Hounsfield units as following**: -**

- A cross-sectional scan of 10-mm thickness will be centered at T11–12 inter vertebral space to image liver and spleen.
- The image will be obtained at end of inspiration by use of 120 kV and a 2-s scan time.
- Liver CT attenuations will be determined by calculating the mean Hounsfield unit (HU) of three regions of interest (ROI) in the liver, and that of spleen also.
- ROI values in the liver and spleen will be selected in peripheral areas away from major portal, arterial, and venous vessels.
- The ratio of liver to spleen (L/S ratio) for CT attenuation values is the index for liver fat degree, with a L/S ratio <1 considered to represent fatty liver.
- **Treatment procedures:**
  - **Diet maintenance**

All patients in both groups (A & B) will follow a regular balanced hypocaloric diet that provided 1700 to 2200 kcal daily during the study period, according to the requirement of each participant. The diet will vary according to each participant's age and eating habits. The diet will be low in fat (20 to 25 %), high in complex carbohydrates (50 to 60%), and sufficient in protein (25 to 30%). No vitamins or other nutritional supplements will be prescribed.

- - **Exercise**

All patients in both groups (A & B) will follow an aerobic exercise 3 to5 times/week for 12 weeks in the form of walking on treadmill for 30 minutes with moderate intensity (moderate intensity = 12-14 according to Borg Scale). Patients will start exercise with 5 mints of warming up and finish with 5 mints of cooling down.

- - **Focused Ultra Sound treatment:**

Focused Ultra Sound using a device of **Mabel6 DUO Ultra Cavitation** will be applied for all patients in group (A) on the abdominal region.

The treatment session will be applied by putting the patient in a comfortable supine lying position and setting the transducer in circular movement that was already secured with conduction gel on the abdominal region which extended bilaterally from the line extending from mid-axilla to iliac crest and above from center of diaphragm to the line extending between two iliac crests below. The same abdominal area will be treated for 30 minutes per session once/ a week for 3 months as a total of 12 sessions.

**Instructions for patients:**

Each subject in both groups (A & B) will be instructed to drink plenty of water during the period of the study to maintain normal body hydration.

All participants will be asked to avoid products that might change the appearance of the skin, such as retinoids and vitamin creams. Additionally, topical steroids will not used on the treatment site for 8 weeks before the treatment and throughout the study to avoid the effect of topical steroids on the inflammatory responses to ultrasound cavitation after the treatment and to assess if there were any complications during and after the application of ultrasound cavitation.

***Statistical analysis:-***

The collected date will be tabular and analyzed using mean and standard deviation and percentage analytical test including paired and unpaired T test for comparing of means between before and after treatment in each group and between groups. P value < 0.05 was accepted as statistical value.

**References**

- **Abenavoli Ludovico, Natasa Milic, Laura Di Renzo, Tomislav Preveden, Milica Medić-Stojanoska, Antonino De Lorenzo (2016).** Metabolic aspects of adult patients with nonalcoholic fatty liver disease. *World J Gastroenterol 2016 August 21; 22(31): 7006-7016.*
- **Akagiri Satomi, Yuji Naito, Hiroshi Ichikawa, Katsura Mizushima, Tomohisa Takagi, Osamu Handa, Satoshi Kokura and Toshikazu Yoshikawa (2008).** A Mouse Model of Metabolic Syndrome; Increase in Visceral Adipose Tissue Precedes the Development of Fatty Liver and Insulin Resistance in High-Fat Diet-Fed Male KK/Ta Mice. J. *Clin. Biochem. Nutr., 42, 150–157, March 2008.*
- [**Al-Dayyat HM**](https://www.ncbi.nlm.nih.gov/pubmed/?term=Al-Dayyat HM%5BAuthor%5D&cauthor=true&cauthor_uid=29571977)**,** [**Rayyan YM**](https://www.ncbi.nlm.nih.gov/pubmed/?term=Rayyan YM%5BAuthor%5D&cauthor=true&cauthor_uid=29571977)**,**[**Tayyem RF**](https://www.ncbi.nlm.nih.gov/pubmed/?term=Tayyem RF%5BAuthor%5D&cauthor=true&cauthor_uid=29571977) **(2018).** Non-alcoholic fatty liver disease and associated dietary and lifestyle risk factors. [*Diabetes Metab Syndr.*](https://www.ncbi.nlm.nih.gov/pubmed/29571977)*2018 Mar 16. pii: S1871-4021(18)30038-9.*
- **Alligier Maud, Laure Gabert, Emmanuelle Meugnier, Ste´ phanie Lambert-Porcheron, Emilie Chanseaume, Frank Pilleul, Cyrille Debard, Vale´ rie Sauvinet, Be´ atrice Morio, Antonio Vidal-Puig, Hubert Vidal and Martine Laville (2013).** Visceral Fat Accumulation During Lipid Overfeeding Is Related to Subcutaneous Adipose Tissue Characteristics in Healthy Men*. J Clin Endocrinol Metab 98: 802– 810, 2013.*
- **Friedmann D. P., Mathew M Avaram, Steven R Cohen, Diane-Duncan, Mitchel P Goldman and Leroy Young (2014).** An evaluation of the patient population for aesthetic targeting abdominal subcutaneous adipose tissue. *Journal of Cosmetic Dermatology, 13, 119- 124.*
- **Gadsden Ernesto, Maria Teresa Aguilar, Bruce R. Smoller and Mark L. Jewell (2011).** Evaluation of a novel High Intensity Focused Ultrasound device for ablating subcutaneous adipose tissue for non invasive body contouring. *Aesthetic surgery journal 31(4) 401- 410.*
- **Janiszewski Peter M., Kevin C., Timothy S. Church, Andrea Dunn, Debra Eshelman, Ronaldo Victor and Robert Ross (2007).** Abdominal obesity, liver fat and muscle composition in survivors of childhood acute lymphoblastic leukemia. *The Journal of Clinical Endocrinology& Metabolism, October 2007, 92(10): 3816- 3821.*
- **Jewel Mark L., Richard A. Baxter, Lisa M. Donofrio, Michael C. Kane, Joel Schlessinger (2012).** Safety and Tolerability of High-Intensity Focused Ultrasonography for Noninvasive Body Sculpting: 24-Week Data from a Randomized, Sham-Controlled Study**.** *Aesthetic Surgery Journal (2012) 32(7) 868–876***.**
- **Jung Un Ju and Myung-Sook Choi (2014).** Obesity and Its Metabolic Complications: The Role of Adipokines and the Relationship between Obesity, Inflammation, Insulin Resistance, Dyslipidemia and Nonalcoholic Fatty Liver Disease. *Int. J. Mol. Sci. 2014, 15, 6184-6223.*
- **Kennedy JE, TerHaar GR, Cranston D (2003).** High intensity focusedultrasound: surgery of the future. Br J Radiol 76(909):590_599.
- **Kennedy J., S. Verne, R. Griffith, L. Falto-Aizpurua, K. Nouri (2015).** Non-invasive subcutaneous fat reduction: a review. *European Academy of Dermatology and Venereology 2015, 29, 1679–1688.*
- **Kim Young Jae, Ji Won Park, Jong Wan Kim, Chan-Soo Park, John Paul S Gonzalez, Seung Hyun Lee, Kwang Gi Kim and Jae Hwan Oh (2016).** Computerized Automated Quantification of Subcutaneous and Visceral Adipose Tissue From Computed Tomography Scans: Development and Validation Study. *JMIR Med Inform 2016; 4(1):e2.*
- **Koska J., Stefan N., Permana P. A., Weyer C. , Sonoda M., Bogardus C., Smith S. R., Joanisse D. R., Funahashi T., Krakoff J., and Bunt J. C. (2008).**Increased fat accumulation in liver may link insulin resistance with Subcutaneous abdominal adipocyte enlargement, visceral adiposity, and hypoadiponectinemia in obese individuals*. Am J Clin Nutr 2008; 87:295–302.*
- **Kwak [Joo Hee,](https://www.sciencedirect.com/science/article/pii/S0261561417303011#!)** **[Dae Won Jun](https://www.sciencedirect.com/science/article/pii/S0261561417303011#!)****, [Seung Min Lee](https://www.sciencedirect.com/science/article/pii/S0261561417303011#!)****, [Yong Kyun Cho](https://www.sciencedirect.com/science/article/pii/S0261561417303011#!)****, [Kang Nyeong Lee](https://www.sciencedirect.com/science/article/pii/S0261561417303011#!)****, [Hang Lak Lee](https://www.sciencedirect.com/science/article/pii/S0261561417303011#!)****, [Oh Young Lee](https://www.sciencedirect.com/science/article/pii/S0261561417303011#!)****, [Ho Soon Choi](https://www.sciencedirect.com/science/article/pii/S0261561417303011#!)** **and [Byung Chul Yoon](https://www.sciencedirect.com/science/article/pii/S0261561417303011#!) (2017).** Lifestyle predictors of obese and non-obese patients with nonalcoholic fatty liver disease: A cross-sectional study. *Journal of Clinical Nutrition.* [*https://doi.org/10.1016/j.clnu.2017.08.018*](https://doi.org/10.1016/j.clnu.2017.08.018)*.*
- **Lee Dong Ho (2017).** Imaging evaluation of non-alcoholic fatty liver disease: focused on quantification. *Clinical and Molecular Hepatology 2017; 23:290-301.*
- **Liu Jiankang, Caroline S. Fox, DeMarc Hickson, Aurelian Bidulescu, J. Jeffery Carr, Herman A. Taylor (2011).** Fatty Liver, Abdominal Visceral Fat, and Cardiometabolic Risk Factors. *Arterioscler Thromb Vasc Biol. 2011; 31:2715-2722.*
- **Lonardo Amedeo, StefanoBallestri, Giulio Marchesini, Paul Angulo and Paola Loria (2015).** Nonalcoholic fatty liver disease: A precursor of the metabolic syndrome. *Digestive and Liver Disease 47 (2015) 181-190.*
- **Milić Sandra, Davorka Lulić, Davor Štimac (2014).** Non-alcoholic fatty liver disease and obesity: Biochemical, metabolic and clinical presentations. *World J Gastroenterol 2014 July 28; 20(28): 9330-9337.*
- **Moreno-Moraga J, Valero-Altés T, Riquelme AM, Isarria-Marcosy MI, dela Torre JR (2007).** Body contouring by non-invasive transdermal focusedultrasound. Lasers Surg Med 39: 315-323.
- **Riechman** **Steven E., Robert E. Schoen, Joel L. Weissfeld, F. Leland Thaete and Andrea M. Kriska (2002).** Association of physical activity and visceral adipose tissue in older women and men. *OBESITY RESEARCH Vol. 10 No. 10 October 2002.*
- **Stephan PJ, Kenkel JM (2010).** Updates and advances in liposuction. *Aesthetic Surg J.; 30:83_97.*
- **Sugun Lee, Hee-Jin Kim, Hyun Jun Park, Hyoung Moon Kim, So Hyun Lee, Sung Bin Cho (2017).** Morphometric analysis of high-intensity focused ultrasound-induced lipolysis on cadaveric abdominal and thigh skin*. Lasers Med Sci (2017) 32:1143–1151.*
- **TerHaar GR, Coussios C (2007).** High intensity focused ultrasound: physicalprinciples and devices. Int J Hyperthermia.; 23(2):89_104.
- **Yunjuan Gu, Haoyong Yu, Yuehua Li, Xiaojing Ma, Junxi, Weihui Yu and Weiping Jia (2013).** Beneficial effects of an 8-week, very low carbohydrate diet intervention on obese subjects*. Evidence Based Complementary and Alternative Medicine, Volume 2013, Article ID 760804, 8 pages.*
